# Immunoglobulin G4-associated autoimmune hepatitis with peripheral blood eosinophilia: a case report

**DOI:** 10.1186/s12876-020-01559-7

**Published:** 2020-12-11

**Authors:** Arunchai Chang, Cheep Charoenlap, Keerati Akarapatima, Attapon Rattanasupar, Varayu Prachayakul

**Affiliations:** 1 Division of Gastroenterology, Department of Internal Medicine, Hatyai Hospital, Songkhla, Thailand; 2 Department of Anatomical Pathology, Hatyai Hospital, Songkhla, Thailand; 3 Siriraj Gastrointestinal Endoscopy Center, Division of Gastroenterology, Department of Internal Medicine, Siriraj Hospital, Faculty of Medicine, Mahidol University, Bangkok, 10700 Thailand

**Keywords:** Immunoglobulin G4 associated autoimmune hepatitis, Autoimmune hepatitis, Immunoglobulin G4 related disease, Eosinophilia

## Abstract

**Background:**

Immunoglobulin G4 (IgG4) associated autoimmune hepatitis (AIH) has been recognized as a type of autoimmune disease that responds to corticosteroid. The diagnosis is based on elevation of the serum IgG4 level, abundance of IgG4 enhanced plasma cell infiltration in the portal region of the liver, and satisfaction of the criteria for “definite AIH” under the revised International Autoimmune Hepatitis Group (IAIHG) scoring system. However, the clinical course of the disease is unclear.

**Case presentation:**

A 65-year-old man with jaundice and peripheral blood eosinophilia. His IAIHG and simplified score was compatible with definite AIH and his IgG4 level was elevated. Magnetic resonance imaging did not reveal abnormalities in the hepatobiliary system or pancreas. A liver biopsy revealed interface hepatitis with IgG4 positive plasma cell infiltration in the portal region, without evidence of bile duct injury. He responded to 4-week period of induction prednisolone therapy and had no recurring symptoms under maintenance therapy of 5 mg prednisolone during the 3-year follow up.

**Conclusions:**

This was a rare case that demonstrated an association between IgG4 associated AIH and the presence of peripheral blood eosinophilia.

## Background

Autoimmune hepatitis (AIH) is an inflammatory liver disease characterized by chronic inflammation of the liver, positivity for autoantibodies, increased immunoglobulin level, and histological evidence of interface hepatitis and lymphoplasmacytic infiltration [1]. In contrast, immunoglobulin G4 (IgG4)-related disease is a chronic, relapsing, systemic, fibro-inflammatory syndrome of presumed autoimmune etiology with high blood levels of IgG4, IgG, and IgE [2]. The association between high serum IgG4 level and high peripheral eosinophil count has been proved; peripheral blood and tissue eosinophilia have been observed in some IgG4-related-disease patients [3]. Recently, AIH with an elevated serum IgG4 concentration and an abundance of IgG4-positive plasma cell infiltration in the liver was proposed to be termed “IgG4 associated AIH” and regarded as a subtype of IgG4-related disease [4]. However, there are few reported cases of IgG4 associated AIH (IgG4-AIH), and the clinical course of this disease remains poorly understood. Here, we report an interesting case of a patient with peripheral blood eosinophilia who was diagnosed with IgG4-AIH and review the literature on this topic.

## Case presentation

A 64-year-old man presented with a 2-month history of progressive, painless jaundice, loss of appetite, and a recent weight loss of 6-kg. He reported a history of uninvestigated, self-limited jaundice with prodromal symptoms occurring 2 years before. His past medical history was negative for peripheral mononeuropathy as well as allergic disease, including allergic rhinitis, sinusitis, and bronchial asthma. He was a non-smoker and did not consume alcohol/supplements/herbals. He also denied previous or ongoing use of drugs. Physical examination was unremarkable except for signs of jaundice and body itching. Complete blood count findings were remarkable for an elevated absolute eosinophil count (AEC) of 2592 cells/μL. Blood chemistry findings included serum total protein, 11.71 g/dL; serum albumin, 2.54 g/dL; total bilirubin, 8.10 mg/dL; direct bilirubin, 6.48 mg/dL, serum aspartate aminotransferase (AST), 385 IU/L; alanine aminotransferase (ALT), 429 IU/L; alkaline phosphatase (ALP), 123 IU/L; and prothrombin time-international normalized ratio, 1.11. Serologic tests were negative for infection with Human Immunodeficiency Virus as well as hepatitis A, B, C or E. The patient’s autoantibody testing (using indirect immunofluorescent method) showed anti-nuclear antibody (ANA) was positive with titer at 1:80 and cytoplasmic pattern while smooth muscle antibody (SMA), anti-mitochondrial antibody and anti-neutrophil cytoplasmic antibodies were negative. The patient’s serum IgG concentration level was 5222 mg/dL (reference range: 548–1768 mg/dL), and his IgG4 concentration level was 1780 mg/dL (reference range: 3.9–86.4 mg/dL). Magnetic resonance imaging (MRI) revealed no abnormalities in the hepatobiliary system or pancreas.

**Fig. 1:**
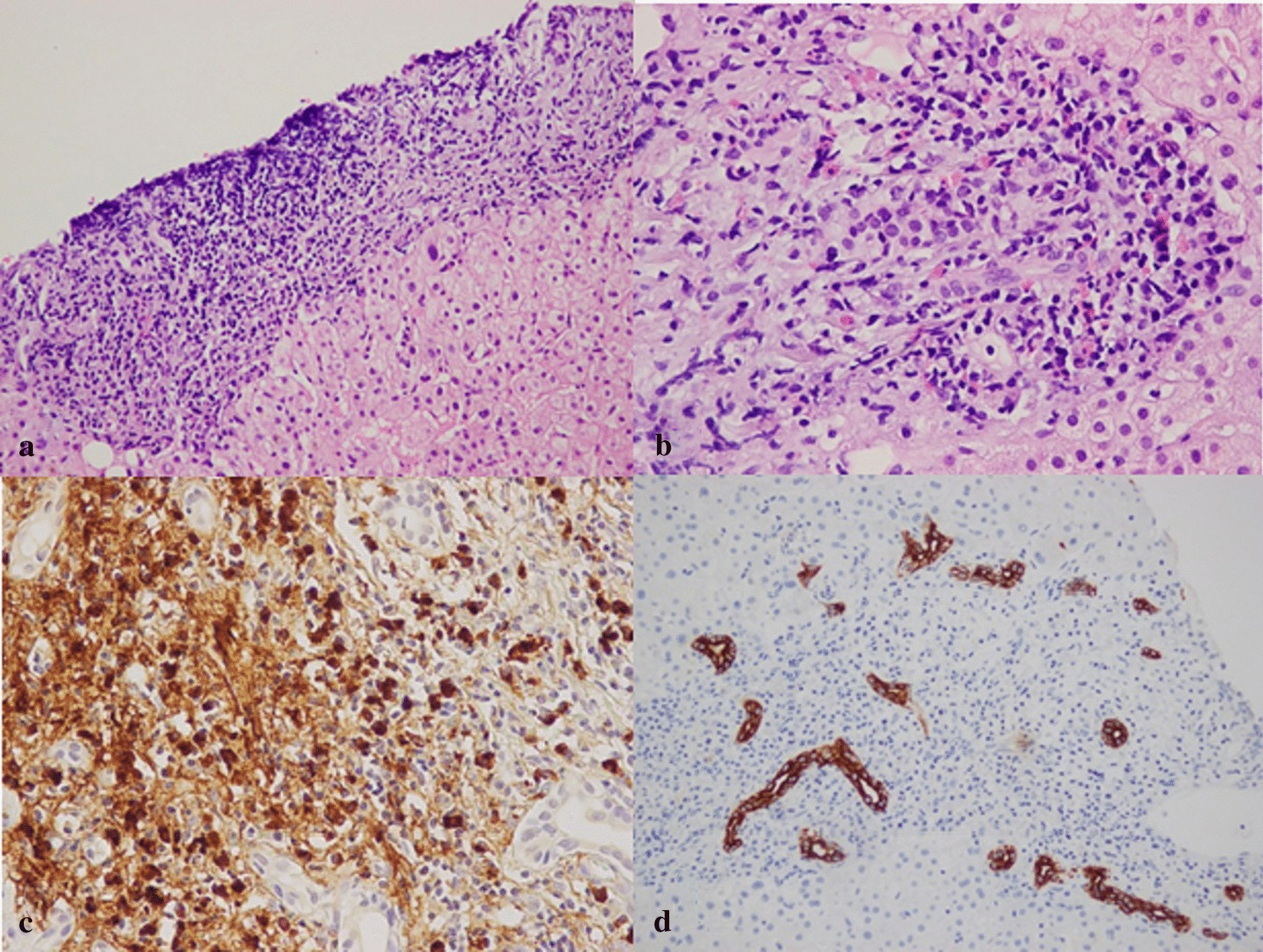
Photographs of liver biopsy demonstrates (**a**) The portal tract expanded by severe inflammatory cell infiltration, consisting of lymphocytes and plasma cells (H&E × 100); (**b**) A moderate number of eosinophils and histiocytes, with interface hepatitis and bridging necrosis, are present; no florid duct lesion or broad collapse is clearly seen (H&E × 400); (**c**) Immunostaining for IgG4 demonstrates that most plasma cells are marked with IgG4, with more than 40 positive plasma cells/high power field; (**d**) Immunohistochemical staining with CK7 revealed a moderate number of central and peripheral bile ducts without evidence of destruction (lumen × 200)

A percutaneous ultrasound-guided liver biopsy was performed (Fig. 1). The pathology results revealed interface hepatitis and bridging necrosis together with severe lymphoplasmacytic cell infiltration, of which most were positive for IgG4 immunostaining (more than 40 IgG4-positive plasma cells/ high power field) in the portal region, with a ratio of IgG4/IgG positive plasma cells greater than 40%. Eosinophilic infiltration was detected with more than 10 cells/high power field. No evidence of bile duct destruction, granuloma, broad collapse or necrotizing vasculitis was noted. Definite AIH was diagnosed based on the patient’s pretreatment revised International Autoimmune Hepatitis Group (IAIHG) score of 17 [5] and the simplified AIH score of 8 [6]. Peripheral blood eosinophilia was diagnosed based on an elevated absolute eosinophil count more than 500cells/μL. Initially, the patient was successfully treated with 40 mg of prednisolone daily for 4 weeks. At his 4-week follow-up visit, the patient exhibited no signs of jaundice, and his liver function tests, and blood eosinophil count eventually normalized. After steroid dose tapering, he continued taking prednisone 5 mg daily to prevent disease recurrence. He decided to receive lifelong prednisolone without undergoing second liver biopsy for disease activity evaluation. His illness did not recur under maintenance therapy of 5 mg prednisolone during the 3-year follow up. His main laboratory results at presentation and during treatment are shown in Table 1.

**Table 1:** Laboratory data at presentation and during treatment

Laboratory	At presentation	At 4 weeks after treatment	At 8 weeks after treatment	At 3 years after treatment	Reference range
Total protein (g/dL)	11.71	6.32	7.80	7.42	5.7–8.2
Albumin (g/dL)	2.54	3.52	4.19	4.66	3.2–4.8
Total bilirubin (mg/dL)	8.10	0.5	0.7	0.6	0.3–1.2
Conjugated bilirubin (mg/dL)	6.48	N/A	N/A	N/A	< 0.2
AST (IU/L)	385	26	15	22	< 34
ALT (IU/L)	429	60	22	27	10–49
ALP (IU/L)	123	110	105	111	45–129
INR	1.11	1.05	0.98	0.99	
IgG (mg/dL)	5222	N/A	N/A	N/A	548–1768
IgG4 (mg/dL)	1780	N/A	274	N/A	3.9–86.4
Total white blood count (cells/μL)	8640	12,640	13,720	9850	4500-10,000
Eosinophil blood count (cells/μL)	2592	10	270	180	

*AST* Aspartateaminotransferase, *ALT* Alanine aminotransferase, *ALP* Alkaline phosphatase, *INR* International normalized ratio, *IgG* immunoglobulin G, *IgG4* immunoglobulin G4, *N/A* Not available

## Discussion

The IgG4-related disease has recently been defined as a systemic, chronic, relapsing, multiorgan, fibroinflammatory condition. It commonly involves the pancreas, biliary tract, salivary glands, lacrimal glands, and kidneys [2]. The hepatic manifestations of IgG4-related disease are heterogenous and remain unclear [7].

However, it has recently been postulated that there is an association between IgG4-related disease and autoimmune hepatitis, and this has given rise to the disease concepts known as “IgG4 hepatopathy” [4] and “IgG4 associated autoimmune hepatitis” [8]. According to Nakanuma et al., these two types of IgG4-related liver disease can be distinguished clinicopathologically [9]. IgG4 hepatopathy is a collective term for hepatic lesions primarily or secondarily related to sclerosing cholangitis and autoimmune pancreatitis; relevant histological findings include portal inflammation, interface hepatitis, lobular hepatitis, large bile duct obstruction, and canalicular cholestasis [10]. In contrast, IgG4-AIH is clinicopathologically similar to AIH except for marked infiltration of IgG4-positive plasma cells in the liver tissue and elevated serum levels of IgG4 [8]. However, agreed-upon diagnostic criteria have not yet been established. The previously published literature indicates that an IgG4-AIH diagnosis may be based on three features: (1) a finding of “definite AIH” according to the IAIHG scoring system, (2) a serum IgG4 concentration level of at least 135 mg/dL, and (3) an IgG4-expressing plasma cell infiltration of at least 10 cells/high power field in the portal tract [8]. Our patient met all these criteria: his IAIHG score indicated “definite AIH”; his IgG4 level was high, and he had an IgG4/IgG-positive cell ratio greater than 40%. In addition, the findings typical of pancreatic lesion/sclerosing cholangitis were not seen on MRI, nor was there any evidence of portal sclerosis or liver cholestasis. For these reasons, the patient was diagnosed with IgG4-AIH and not IgG4 hepatopathy.

An important question is whether IgG4-AIH is a subtype of classic AIH or a subtype of IgG4-related disease that involved liver. Previous reports suggest that IgG4-AIH should be differentiated from classic AIH [8]. Most baseline biochemical and autoantibody laboratory values do not help distinguish between IgG4-AIH and classic AIH. In contrast, serum concentration levels of both IgG and IgG4 are significantly higher in patients with IgG4-AIH than in those with classic AIH [8, 11]. Histological finding of eosinophilic infiltrate is not a characteristic feature of IgG4-AIH because this could be detected in patients with both group [8]. Both conditions respond dramatically to glucocorticoid therapy. The long-term response to glucocorticoid therapy is comparable for each situation; however, the alanine aminotransferase normalization time after initiation of such treatment is shorter in IgG4-AIH patients [10]. Chung et al. proposed that the degree of accumulation of IgG4-positive liver cells is associated with the serum IgG4 response in patients with IgG4-AIH [11]. Furthermore, synchronous or metachronous development of other IgG4-related disease is observed in most cases of IgG4-AIH [9, 12–15]. This evidence suggests that IgG4-AIH is a hepatic manifestation of IgG4-related disease and, while sharing pathological findings with AIH, is not a subtype of classic AIH [9]. To our knowledge, this is the second reported case of IgG4-AIH, which had only hepatitis after those reported by Umemura et al. [16].

Another important aspect of our case was the presence of peripheral blood eosinophilia and the abundance of eosinophilic infiltration in the biopsied liver specimen. IgG4 and IgE share a common immune response pathway. Allergic immunology triggers T-helper 2-type immune response that promotes secretion of IgG4 and IgE and induces peripheral blood and tissue eosinophilia [17]. There are data supporting a positive association between IgG4 and both IgE and peripheral eosinophil count [18]. Further, peripheral blood and tissue eosinophilia have been observed in some cases of IgG4-related disease [8]. Moreover, the histologic features of IgG4-related disease often involve increased eosinophil infiltration [2]. Although serum IgE levels were not obtained in this case, our finding of peripheral blood and tissue eosinophilia could be explained by the presence of IgG4-related disease. In a recent study by Mohapatra et al. [3], it was shown that peripheral eosinophilia increased as serum IgG4 increased. This finding was consistent with a recently reported case of IgG4-AIH with the laboratory of IgG4 level is remarkably high (3560 mg/dL) presenting as idiopathic hypereosinophilia syndrome (AEC 17,940 cells/μL) [19]. The author concluded that having peripheral eosinophilia may correspond with having a higher level of serum IgG4. These data are consistent with our findings: our patient had an extremely high level of serum IgG4 (1780 mg/dL) along with significant peripheral eosinophilia (an absolute eosinophil count of 2592 cells/μL). However, the utility of peripheral eosinophilia as a tool for diagnosing IgG4-related disease requires further investigation.

In conclusion, we report a rare case of IgG4-AIH with peripheral blood eosinophilia and absence of symptoms related to other organ of IgG4-related disease. The identification of “Definite AIH” based on both IAIHG and simplified AIH score, high serum IgG4 level, and an accumulation of IgG4-positive cells in the liver were necessary for our diagnosis. Glucocorticoid treatment is the first line treatment for this condition and, in our patient, produced a good response. Although the presence of peripheral eosinophilia may possibly help diagnose this disease, further studies are needed to confirm this and to clarify the clinical course and optimal treatment of IgG4-AIH.

## Data Availability

The datasets used and analyzed during the current study are available from the corresponding author on reasonable request.

## Abbreviations

AIH: Autoimmune hepatitis
IgG4: Immunoglobulin G4
IgG4-AIH: Immunoglobulin G4associated AIH
AST: Aspartate aminotransferase
ALT: Alanine aminotransferase
IAIHG: International Autoimmune Hepatitis Group
